# As right as rain: deciphering drought-related metabolic flexibility in the C_4_–CAM *Portulaca*

**DOI:** 10.1093/jxb/erac179

**Published:** 2022-08-11

**Authors:** Ivan Reyna-Llorens, Sylvain Aubry

**Affiliations:** Centre for Research in Agricultural Genomics (CRAG) CSIC-IRTA-UAB-UB, Campus UAB, Bellaterra, Barcelona, Spain; Department of Plant and Microbial Biology, University of Zürich, 8008 Zürich, Switzerland

**Keywords:** C_4_, CAM, photosynthesis, *Portulaca oleracea*

## Abstract

This article comments on:

**Ferrari RC, Kawabata AB, Ferreira SS, Hartwell J, Freschi L.** 2022. A matter of time: regulatory events behind the synchronization of C_4_ and crassulacean acid metabolism gene expression in *Portulaca oleracea*. Journal of Experimental Botany **73,**4867–4885.

**Optimization of carbon and water usage in plants is a widespread strategy to survive in hot and dry environments. Based on the ancestral C_3_ photosynthesis, two major carbon -concentrating mechanisms (CCMs) evolved, allowing spatial (C_4_) or temporal (Crassulacean acid metabolism, CAM) segregation of carbon-fixing activities. While C_4_ and CAM have generally been considered mutually exclusive, they share most of their biochemical machinery. An exception to the rule is found among the Caryophyllaceae, in the *Portulaca* genus. Described as an ‘inducible’ CAM, the C_4_ species *Portulaca oleracea* accumulates malate transiently under drought stress and in a reversible manner. Concentrating mostly at the transcriptome level, Ferrari *et al*. try deciphering the complex interplay between C_4_ and CAM under various drought conditions. A better understanding of how the two carbon-fixing mechanisms are coordinated could shed light on key regulatory mechanisms necessary to improve C_4_ crops under changing environments.**

Most of the enzymatic machinery that is required to run C_4_ and CAM cycles has derived from ancestral C_3_ species, where these enzymes mostly played anaplerotic roles (Silvera *et al.*, 2010; Aubry *et al.*, 2011). Both C_4_ and CAM are impressive examples of convergent evolution, with at least 66 and 40 independent origins, respectively (Silvera *et al.*, 2010; Sage *et al.*, 2012). Typically, in C_4_, a subset of proteins is limited to bundle sheath or mesophyll cells, allowing concentrating of CO_2_ around the central carboxylase Rubisco, thus reducing the penalties of photorespiration (Fig. 1). While in species running CAM, carbon fixation by a phospho*enol*pyruvate carboxylase (PEPC) is transposed into the dark phase, connected to malate accumulation, its efflux from the vacuole and subsequent decarboxylation take place during the day. Interestingly, both CAM and C_4_ not only enable more efficient carbon fixation, but also generally improve water use efficiency. The inverse pattern of stomatal opening in CAM species is primarily aimed at limiting water loss during the light phase, while C_4_ leaves operate at lower stomatal conductance compared with C_3_ (Aubry *et al.*, 2016; Males and Griffiths, 2017). It is noteworthy that within both CAM and C_4_ species, a whole range of non-canonical adaptations evolved, moving away from ‘prototypical’ models, for example CAM species still fixing carbon on a 24 h basis or various C_3_–C_4_ intermediates (Owen and Griffiths, 2013; Schlüter and Weber, 2020).

**Fig. 1.**
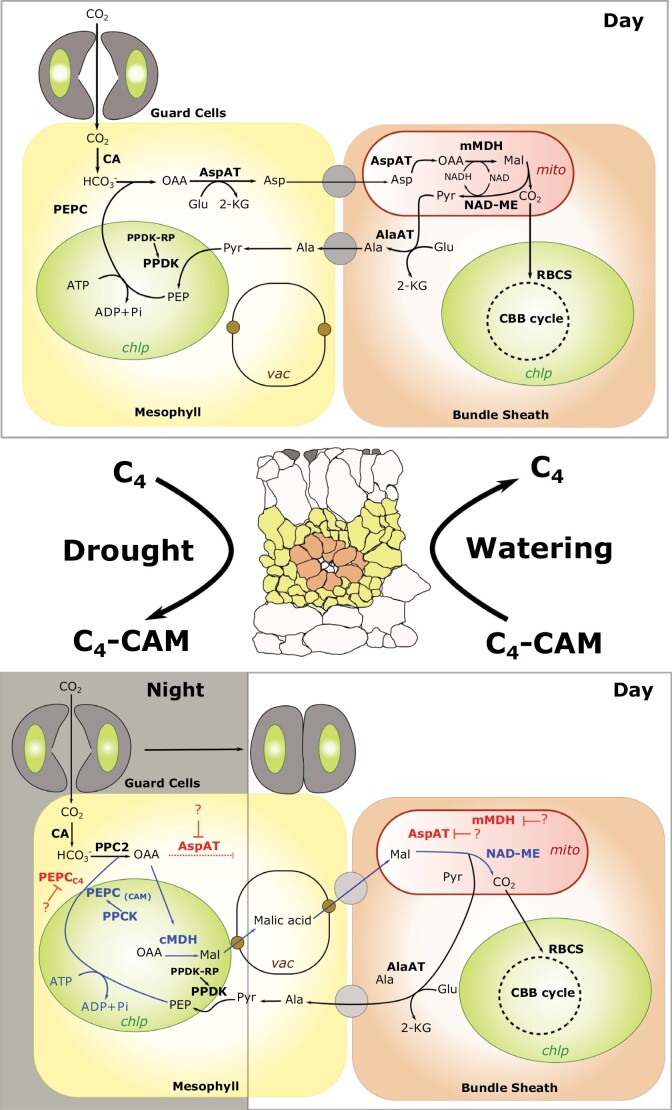
Working model illustrating the C_4_–CAM switch in the NAD-ME-type C_4_*Portulaca oleracea*. A transversal section of a *P. oleracea* leaf is illustrated in the middle, guard cells in grey, mesophyll cells in yellow, and bundle sheath cells in orange. Enzymes highlighted in red represent those C_4_ enzymes that need to be down-regulated or repressed for the activation of the CAM cycle. At the same time, enzymes in blue denote those enzymes that would have to be activated or modified during CAM induction. Abbreviations: Chlp, chloroplasts are in green; mito, mitochondria in red; vac, vacuole; CA, carbonic anhydrases; PEPC, phospho*enol*pyruvate carboxylase; AspAT, aspartate amino acid transferase; AlaAT, alanine amino acid transferase; NAD-ME, NAD-dependent malic enzyme; PPDK, pyruvate.orthophosphate dikinase; PPDK-RP, PPDK regulatory protein; RBCS, Rubisco; mMDH, mitochondrial malic dehydrogenase; cMDH, chloroplastic malic dehydrogenase; PPCK, PEPC kinase; Mal, malate; Ala, alanine; Pyr, pyruvate; Glu, glutarate; 2-KG, 2-ketoglutarate; OAA, oxaloacetate.

Consideration of this flexibility is important when thinking in terms of engineering C_4_ in a C_3_, CAM in a C_3_, or, our focus here, CAM in a C_4_ (Box 1). Several anatomical, physiological, and biochemical barriers have led to the assumption that CAM and C_4_ photosynthesis were incompatible (Sage, 2002). In particular, concomitant mesophyll (C_4_-)carboxylation and (CAM-)decarboxylation may result in futile cycles. Meanwhile, at least three genera primarily using C_4_ photosynthesis (namely *Portulaca*, *Spinifex*, and *Trianthema*) present temporary acidification and reversed stomatal behaviour under drought stress, signatures of CAM (Gilman *et al.*, 2022). The *Portulaca* family belongs to the Caryophyllales, where eight of the 23 families encompass C_4_ and C_3_–C_4_ species (Voznesenskaya *et al.*, 2010). The peculiar metabolic flexibility of *Portulaca oleracea*, a facultative CAM species using an NAD-malic enzyme (ME)-type C_4_ metabolism, is a good model to study interplays between various transcriptional, translational, and metabolic regulations. Interestingly, despite strong anatomical constraints, at the biochemical level CAM inception appears to be more flexible (and reversible) than the C_4_ pathway: while there is no such thing as a facultative/inducible C_4_ pathway, there are some examples of inducible CAM in both C_3_ and C_4_ backgrounds (Wai *et al.*, 2019; Ferrari *et al.*, 2022; Gilman *et al.*, 2022). Engineering CAM into naturally non-CAM crops may improve water use efficiency and stress resilience (Schiller and Bräutigam, 2021).

**Box 1. Bringing microgenomics into CAM research**

The complexity of the metabolic interplay and diversity in C_4_–CAM species ran into several limitations that may be alleviated by recent advances in molecular biology and genomics. In the C_4_ field, where the cell-specific component might have been more obvious, research efforts have been undertaken to unravel evolutionary, biochemical, and metabolic complexity (Schlüter and Weber, 2020). While phylogenomics approaches have provided compelling results towards the origins of the CAM syndrome and its plasticity, only few recent works address CAM using microgenomics (Abraham *et al.*, 2016). Such an approach will be made easier by the sequencing of genomes from CAM species and may allow monitoring of steady-state transcript abundance, and cell-specific transcriptional and translational regulatory processes in individual cell types that would, in turn, result in a better understanding of gene regulatory networks underlying C_3_/C_4_–CAM interplay. For example, whether and how water storage cells present in many succulent species (e.g. *P. oleracea*) are influencing the C_4_ cycle, or the extent to which diurnal malate pools in the mesophyll are influencing the rhythmicity of stomatal apertures remain open questions that may only be addressed using cell-specific approaches. A more precise description of the C_4_–CAM switch that reflects the actual plasticity of the CAM syndrome is required: not all plants could be switched into CAM, not all species switch in a similar time frame (Dodd *et al.*, 2002), and, finally, not all cells may adapt their metabolism to the same extent.

## CAM and the circadian clock

In their contribution, Ferrari and colleagues evaluate the extent to which the underlying circadian clock regulation might modulate the shift to temporal gene expression under CAM induction by drought stress. Generally, studies on obligate CAM (*Mesembryanthemum crystallinum*, *Kalanchoe fedtschenkoi*, and *K. laxiflora*) show that circadian clock elements are mostly unaffected by drought, in terms of both phasing and amplitude. Nevertheless, diurnal variations appear to be a necessary requirement to ensure proper CAM-related metabolic fluxes (Boxall *et al.*, 2020). In recent years, our understanding of the complexity of gene circuits responsible for the core clock oscillation improved dramatically (Millar, 2016). Ferrari and colleagues report that none of these genes was significantly affected by drought. Two levels of regulation are possibly coordinating CO_2_ uptake in CAM: the circadian oscillator control and the metabolite control (Dodd *et al.*, 2002). While C_4_ photosynthesis is essentially based on cell differentiation, it is also important to consider the circadian clock not necessarily ‘ticking’ at the same pace in all cells and tissues (Greenwood and Locke, 2020), and therefore not regulating all genes involved in carbon metabolism in the same way in all cells. To unravel the complex metabolic interplay underlying C_4_ to CAM transition in stressed *P. oleracea*, more studies on cell-specific variations of the circadian clock under stress as well as the conservation of gene regulatory networks (specifically *cis-*elements of clock target genes) that are under control of the core clock genes appear very relevant.

## Central role of diurnal PPCK in the C_4_–CAM transition

*Portulaca oleracea* is a bona fide C_4_ species with the capacity of switching from C_4_ to CAM in response to drought stress (Voznesenskaya *et al.*, 2010). By taking advantage of the facultative nature of this species, Ferrari and colleagues assessed the contribution of both the clock and drought in the activation of the transcriptional programme for CAM induction. In *P. oleracea,* transcriptional induction and repression of both CAM and C_4_ genes were mainly affected by drought conditions. On the other hand, while the circadian clock is fundamental for CAM activation, its disruption only affected the expression of *PPCK-E1* among the genes involved in CAM. Phosphoenolpyruvate carboxylase kinase (PPCK) is responsible for activating the CAM carboxylase PEPC that controls the initial fixation of CO_2_ during the night (Hartwell *et al.*, 1999). The influence of both clock and drought on PPCK could be essential for CAM induction and control, acting as a fine-tuning switch that limits PEPC activity to the dark period once CAM has been established. Understanding how clock and drought signals converge in the regulation of PPCK is essential for engineering CAM in both C_3_ and C_4_ species (Box 2).

**Box 2. C_4_–CAM: a physiology still to be explored**

The C_4_–CAM switch appears to be a possible way to complement C_4_-centric carbon concentration, particularly for some of the crops that may encounter increasingly arid conditions in the near future. From an engineering perspective, an inducible CAM system would possibly allow plants to tolerate more extreme environments (where C_4_ plants underperform) while still relying on C_4_ metabolism under optimal conditions. However, in order to achieve this ambitious goal, several gaps in our understanding of the C_4_–CAM interplay need to be addressed. A first point might be to try to understand the actual limits of C_4_ hydraulics towards drought stress and the extent to which succulence may affect metabolic regulation and C_4_ pathways. Secondly, the major change in malate homeostasis would require temporal and spatial changes in gene expression (namely alterations of PEPC, MDH, and NAD-ME) that would necessarily impact the whole tissue (Fig. 1). Down-regulation in C_4_ gene expression upon CAM induction might require rewiring of higher order gene regulatory networks such as the circadian and other light signalling networks. Typically, a proper PPCK activation in time and during drought will be crucial to kick-start carboxylation into the CAM route. A better understanding of these processes is a prerequisite for any attempt considering CAM as a valuable asset for improving crop resilience.

## Hormonal cues involved in the C_4_ to CAM transition

At the transcriptional level, the C_4_–CAM system in *P. oleracea* seems to be triggered by the water status of the plant. Ferrari and colleagues further explored this connection by assessing the activities of abscisic acid (ABA) and cytokinins (CKs). ABA is known to mediate abiotic stress responses in plants and has been associated with CAM expression in other species such as agave or pineapple (Chen *et al.*, 2020). The role of CKs in CAM is less clear, yet some evidence suggests its involvement in CAM induction in the C_3_+CAM *M. crystallinum* (Wakamatsu *et al.*, 2021). Indeed, endogenous levels of ABA in *P. oleracea* increased in correlation with a reduction in osmotic potential and intracellular acidification proper of CAM metabolism. The correlation between ABA metabolism and CAM induction was also observed at the transcriptional level, where ABA biosynthesis genes and several components of the ABA signalling pathway were up-regulated under drought conditions and down-regulated during re-watering. CK genes, on the other hand, showed a more delayed induction during re-watering, suggesting a potential role at later stages of C_4_–CAM induction. In fact, addition of exogenous CK reverted the suppression of C_4_ genes in drought-stressed plants while exogenous ABA triggered transcription of the CAM *PPC1E1c* gene. Despite this, neither ABA nor CK treatments induced a change in intracellular ∆H^+^, implying that ABA is not sufficient to trigger CAM in *P. oleracea*. The exact role of these two phytohormones for the CAM switch remains unclear.

To find potential links between ABA and CK signalling networks and the C_4_–CAM induction in *P. oleracea*, Ferrari and colleagues defined a group of transcription factor candidates based on a gene co-expression network built from previously published transcriptomics data (Ferrari *et al.*, 2020). Indeed, a group of nine transcription factor candidates responded to drought conditions. Similarly, their expression was influenced by the exogenous addition of either ABA or CK, which is consistent with the patterns observed for both CAM and C_4_ PEPC genes. Based on these results, the authors proposed a transcriptional regulatory network for C_4_–CAM induction in *P. oleracea*. This work paves the way for further characterization of the transcription factors as well as the development of *P. oleracea* as a model system to address CAM induction in the C_4_ context, similar to *M. crystallinum* for C_3_+CAM induction (Wakamatsu *et al.*, 2021).

## Perspectives

Ferrari and colleagues take advantage of whole-transcriptome analysis to try to decipher CAM dynamics, its interplay with the C_4_ pathway, and drivers of its induction upon drought stress. While this approach is interesting, a comprehensive view over the C_4_ to CAM transition is still missing. For example, few transcripts actually do match peak enzyme activities, and variation in transcript abundance may not entirely correlate with protein abundance and activity, nor with the actual metabolite signatures (Abraham *et al.*, 2016). Therefore, it would be interesting also to collect proteomics data and test if protein abundance matches the enzyme activities better than transcripts, or if the enzyme regulation relies on further post-translational mechanisms. Recent genome sequencing of *Portulaca amabilis*, a diploid facultative CAM using NADP-type C_4_, identified a specific PEPC orthologue for each carboxylation as well as evidence for cross-linking nocturnal acid production into the C_4_ cycle (Gilman *et al.*, 2022).

The extent to which *P. amabilis* and *P. oleracea* coordinate their carbon fluxes in a comparable way remains to be determined. Data presented here open a whole new perspective over the way the C_4_–CAM switch could have been recruited to bring some fitness improvements in arid conditions for already efficient CCMs. Exploring further the genomic space of *Portulaca* will allow identification of regulatory elements both in cis and *in trans*, and help in deciphering the regulatory network underlying this complex metabolic interplay. As for any other biochemical pathways (e.g. C_4_ acid decarboxylations, Furbank, 2011), metabolism ‘subtypes’ are useful intellectual constructs to try making order out of chaos, but often do not match reality. Above all, these categorizations should not limit us in the quest to understand the fantastic plasticity of plant metabolism.
